# Progress in State Medicine

**Published:** 1903-11-21

**Authors:** 


### Progress in State Medicine.

### HOUSING OF THE WORKING CLASSES.

Williamson[1] states that in Edinburgh much attention has been devoted during 1902 to improving the sanitary conditions of the older properties inhabited chiefly by the working and poorer classes. Dark and unventilated water-closets are being systematically dealt with; 400 new apparatus have been introduced, and 300 apartments structurally altered. The work of measuring and ticketing small houses of a total capacity not exceeding 2,000 cubic feet has been continued. At the end of last year 7,323 small houses had been measured, and on the doors of these tickets were affixed indicating the number of persons for whose accommodation space was provided. Since this work was commenced the results have been very satisfactory; cases of overcrowding steadily decreasing, the total number last year having only reached 244. Special attention has also been devoted to ventilation of common stairs. The necessity for having by-laws for the regulation of farmed-out houses and houses let in lodgings is now much emphasised by these classes of houses being much on the increase in Edinburgh. There is a well-marked distinction between the classes. The former group consists chiefly of large tenements, or portions of tenements, which are leased by house farmers and let out at an exaggerated rent to a very poor class of tenants. The latter group consists of houses which are to all intents and purposes common lodging houses. A population of about 1,800 is housed in the latter. This shows a marked increase in comparison with former years. Strict inquiries have revealed that only in two instances have persons been found resident in any of these houses who had previously been householders within any area affected by operations under an improvement scheme. Matheson [2] draws attention to the fact that during the 60 years, 1841-1901, there was a gradual reduction in the number of houses in Ireland—from 1,328,839 to 858,158. There was also a great alteration in the relative number of houses. In 1841 the number of mud cabins numbered 491,278, forming 36·97 per cent. of the total number of houses. In 1851 the number fell to 135,589, and the percentage to 12·96, whilst in 1901 there were only 9,873, or 1·15 per cent. A large portion of this decrease was due to emigration. Houses of a somewhat better class also showed a falling off, but first- and second-class houses, or good farm houses, increased. In the whole of Ireland in 1841 the number of families having only fourth-class accommodation formed 42·46 of the total families in the country, and in 1901 the percentage was only 4·53. The total number of tenements of one room in Ireland in 1901 was 79,149. Of these there were 20,994 cases in which the room had but one occupant; 41,918 where the room had two, three, or four occupants; 13,351 in which there were five, six, or seven occupants; and 2,886 in which there were eight or more occupants, including 786 cases of nine persons, 364 of ten, 138 of eleven, and 68 of twelve or more persons in the room. Of the 59,265 families in Dublin, 21,747 were located in one-room tenements or 36·70 per cent. Comparing Dublin with other big cities it was found that the number of persons in one-room tenements with five or more occupants in every 100 of the total population was: Dublin, 10·61; Belfast, 0·10; London, 0·70; Liverpool, 0·24; Manchester, 0·05; Edinburgh, 2·33; Glasgow, 5·24. As regards the percentage of the tenements of all classes, which were one room tenements occupied by five or more persons, the three countries stand thus: England, 0·15; Scotland, 3·27; Ireland, 1·78. In England, of a total population of 32,527,843, there were only 60,043 persons, or 0·18 per cent., who were inhabitants of one-room tenements having five or more occupants each. In Scotland the corresponding percentage was 4·20, and Ireland 2·28. Whilst there has been material improvement in the housing of the people of Ireland since 1841, there still remains much to be accomplished. Taylor [3] advocates united municipal action with regard to the housing problem, and also that money should no longer be wasted in curing certain plague spots in the centre, while the surrounding districts were left to grow up in their own haphazard fashion. He would give municipalities a controlling power up to a three-mile limit outside their boundaries. In practice, the dispossessed tenants of slums did not, as a rule, occupy the new houses, firstly, because the rents were too high, and secondly, because demolition frequently took place before the new property was erected. The first duty of a municipality was not to the artisan but to the poorest poor, who were ejected in the general interest of the community. Changes needed in the law might be grouped under such headings as, simplification of procedure, more favourable financial facilities, modification of building regulations, and the rating of vacant sites on their capital value. Turton [4] mentions that the Liverpool Housing Committee had decided that in future new houses should only be occupied by dispossessed tenants, each of whom was given an opportunity of taking a new house. In Liverpool there were in course of erection 176 one-roomed houses (2s. a week), 642 two-roomed (3s.), 738 three-roomed (4s.), and 140 four-roomed (5s); a total of 4,234 rooms to accomodate over 8,000 persons. Taking the total cost of demolition and housing, the loss last year amounted to ½d. in the pound, including sinking funds, but at the end of a period of years the ratepayers would have possession of a valuable rent-producing property.

[1] Pub. Health Engin., June 20, 1903. [2] Dub. Jour. Med. Sci., July, 1903. [3] Pub. Health Engin., Aug. 1, 1903. [4] Ibid.

*(To be continued.)*

